# Supplementary material for: Resolving the full spectrum of human genome variation using Linked-Reads
Source: Genome Res. 2019 Apr;29(4):635–45. doi: 10.1101/gr.234443.118 (PMC6442396; doi:10.1101/gr.234443.118)
Supplement: Supplemental Material [file supp_gr.234443.118_Supplemental_longranger-2.2.zip › longranger-2.2.tar.bz2!/lr2.2-public/lib/stan/cmdstan/stan/doc/api/html/matrix_2min_8hpp_source.html]

Stan: src/stan/math/matrix/min.hpp Source File


|  |  |
| --- | --- |
|  | Stan  2.5.0  probability, sampling & optimization |


- Main Page
- Related Pages
- Namespaces
- Classes
- Files

- File List
- File Members

All Classes Namespaces Files Functions Variables Typedefs Enumerations Enumerator Friends Macros Pages

- src
- stan
- math
- matrix

min.hpp

Go to the documentation of this file.

1 #ifndef STAN\_\_MATH\_\_MATRIX\_\_MIN\_HPP

2 #define STAN\_\_MATH\_\_MATRIX\_\_MIN\_HPP

3

4 #include <stdexcept>

5 #include <vector>

6 #include <stan/math/matrix/Eigen.hpp>

7

8 namespace stan {

9  namespace math {

10

18  inline int min(const std::vector<int>& x) {

19  if (x.size() == 0)

20  throw std::domain\_error("error: cannot take min of empty int vector");

21  int min = x[0];

22  for (size\_t i = 1; i < x.size(); ++i)

23  if (x[i] < min)

24  min = x[i];

25  return min;

26  }

27

35  template <typename T>

36  inline T min(const std::vector<T>& x) {

37  if (x.size() == 0)

38  return std::numeric\_limits<T>::infinity();

39  T min = x[0];

40  for (size\_t i = 1; i < x.size(); ++i)

41  if (x[i] < min)

42  min = x[i];

43  return min;

44  }

45

52  template <typename T, int R, int C>

53  inline T min(const Eigen::Matrix<T,R,C>& m) {

54  if (m.size() == 0)

55  return std::numeric\_limits<double>::infinity();

56  return m.minCoeff();

57  }

58

59  }

60 }

61 #endif

Eigen.hpp

stan::math::min

double min(const double a, const double b)

**Definition:** min.hpp:7


---

[ Stan Home Page ]

*© 2011–2014,
Stan Development Team.*
